# Accuracy of consumer-based activity trackers as measuring tool and coaching device in patients with COPD and healthy controls

**DOI:** 10.1371/journal.pone.0236676

**Published:** 2020-08-04

**Authors:** Astrid Blondeel, Heleen Demeyer, Wim Janssens, Thierry Troosters

**Affiliations:** 1 Department of Rehabilitation Sciences, KU Leuven, Leuven, Belgium; 2 Clinical Department of Respiratory Diseases, University Hospitals Leuven, Leuven, Belgium; 3 Department of Rehabilitation Sciences, Ghent University, Gent, Belgium; 4 Department of Chronic Diseases, Metabolism and Aging (CHROMETA) – BREATHE, KU Leuven, Leuven, Belgium; Curtin University, AUSTRALIA

## Abstract

**Background:**

Consumer-based activity trackers are used to measure and improve physical activity. However, the accuracy of these devices as clinical endpoint or coaching tool is unclear. We investigated the use of two activity trackers as measuring and coaching tool in patients with Chronic Obstructive Pulmonary Disease (COPD) and healthy age-matched controls.

**Methods:**

Daily steps were measured by two consumer-based activity trackers (Fitbit Zip, worn at the hip and Fitbit Alta, worn at the wrist) and a validated activity monitor (Dynaport Movemonitor) in 28 patients with COPD and 14 healthy age-matched controls for 14 consecutive days. To investigate the accuracy of the activity trackers as a clinical endpoint, mean step count per patient were compared with the reference activity monitor and agreement was investigated by Bland-Altman plots. To evaluate the accuracy of activity trackers as coaching tool, day-by-day differences within patients were calculated for all three devices. Additionally, consistency of ranking daily steps between the activity trackers and accelerometer was investigated by Kendall correlation coefficient.

**Results:**

As a measuring tool, the hip worn activity tracker significantly underestimates daily step count in patients with COPD as compared to DAM (mean±SD Δ-1112±872 steps/day; p<0.0001). This underestimation is less prominent in healthy subjects (p = 0.21). The wrist worn activity tracker showed a non-significant overestimation of step count (p = 0.13) in patients with COPD, and a significant overestimation of daily steps in healthy controls (mean±SD Δ+1907±2147 steps/day; p = 0.006). As a coaching tool, both hip and wrist worn activity tracker were able to pick up the day-by-day variability as measured by Dynaport (consistency of ranking resp. r = 0.80; r = 0.68 in COPD).

**Conclusion:**

Although the accuracy of hip worn consumer-based activity trackers in patients with COPD and wrist worn activity trackers in healthy subjects as clinical endpoints is unsatisfactory, these devices are valid to use as a coaching tool.

## Introduction

Physical inactivity has been related to health outcomes such as mortality, hospitalization risk [1] and disease progression [2] in patients with Chronic Obstructive Pulmonary Disease (COPD). Being physically active can provoke symptoms of dyspnea in these patients and these unpleasant symptoms lead to avoidance of physical activity (PA) [3]. A decreased PA is already present early in the disease [4] and PA further declines with disease progression [5]. PA can be objectively measured by using accelerometers, which have been validated in patients with COPD [6, 7].

Nowadays, more consumer-based activity trackers are used [8]. These devices have a lower cost as compared to medical device classified accelerometers, are easy to use and most of them provide direct feedback on PA levels. Depending on the device, these activity trackers can provide activity-related information such as step counts, energy expenditure, intensity of activities and heart rate. Research on the measurement properties of previous generations of waist worn step counters showed that the validity of these step counters was sufficient in healthy subjects, but these waist worn step counters underestimated step count during slower walking [9, 10]. Newer generation of activity trackers (including tri-axial accelerometry) are available now but it remains unclear whether these more advanced activity trackers could be used as a valid way to capture physical activity endpoints in clinical trials in patients with COPD. In addition, it is not known whether the placement of the tracker (wrist or hip worn) influences the measurement properties of these trackers. Wrist worn devices might be more convenient, particularly for direct feedback on PA.

PA promotion is included as a recommended non-pharmacological intervention for all stages of COPD in the GOLD strategy [11]. Several strategies to improve PA have been investigated in patients with COPD [12]. Coaching interventions with use of activity trackers showed significant improvements in PA [13, 14]. By monitoring and providing (real-time) feedback to patients, these step counters or activity trackers play a crucial role in the coaching interventions [15]. A qualitative analysis showed that 76.1% of the patients involved in a tele coaching intervention considered the step counter as the most important part of the intervention [16]. The prerequisite for using these activity trackers to coach individual patients is that they can accurately capture the individual day-by-day changes. For coaching purposes, the device should be capable of distinguishing more and less active days within a subject.

Finally, to accomplish long-term behavior changes with coaching, activity trackers should be user-friendly and subjects should be willing to wear these devices over a longer period of time. Different common placement on the body (i.e. hip or wrist) may have different validity and acceptability.

Therefore, the aim of this study is to investigate the accuracy of two consumer-based activity trackers (i.e. Fitbit Zip, worn at the hip and Fitbit Alta, worn at the wrist) in patients with COPD and healthy age-matched controls, 1) to measure the PA of a patient (‘accuracy as a measuring tool’); and 2) to distinguish more and less active days at an individual level (‘validity as a coaching tool’), as compared to a validated activity monitor (i.e. Dynaport Movemonitor). We hypothesize 1) that activity trackers will not be able to accurately measure daily steps in patients with COPD, and 2) that these activity trackers are capable to distinguish more and less active days within each patient and thus are valid to be used as a coaching tool. Finally, we aim to provide insight in the patients’ preference.

## Methods

### Population and design

In the present study, 30 patients with COPD were included. Patients with a diagnosis of COPD (confirmed by spirometry (FEV_1_/FVC ≤ 0.70)), with an age above 40 years old, with a smoking history of at least 10 pack years and no moderate or severe exacerbations within 4 weeks prior to inclusion were eligible for the present study. Patients were recruited at the University Hospitals Leuven (Leuven, Belgium) between August 2017 and November 2019. In addition, we included 15 age-matched healthy controls who were in absence of any known respiratory disease, without airflow obstruction (confirmed by spirometry) and who had no or a marginal smoking history (< 5 pack years). For both groups, subjects were not included if they used a walking aid in daily life or if they had an impaired gait pattern, as judged by the investigator. The study was approved by the ethical committee of University Hospital Leuven (s60227) and all subjects signed the informed consent prior to data collection. This observational study consisted of one clinical visit and a testing phase of 14 days. During the testing phase, subjects were instructed to wear the activity monitor and two activity trackers simultaneously. At the end of the testing phase, subjects were asked to complete a questionnaire collecting information about the experience of patients with wearing the activity trackers.

### Clinical measurements

At the clinical visit, both groups performed the same clinical assessments: 1) an anthropometric assessment (weight and height); 2) a post-bronchodilator spirometry (according to the ATS-ERS guidelines) retrieving FEV_1_ and FVC [17]; 3) functional exercise capacity measured by the best out of two six-minute walking tests conducted in a 50 m corridor using standardized encouragement [18]; 4) symptoms of dyspnea assessed by the modified Medical Research Council (mMRC) dyspnea scale [19]; 5) clinical visit version of PROactive questionnaire, investigating patient’ reported physical activity experience [20]; and 6) health status questioned by the COPD Assessment Test (CAT) [21].

### Physical activity monitor

**Fig 1:**
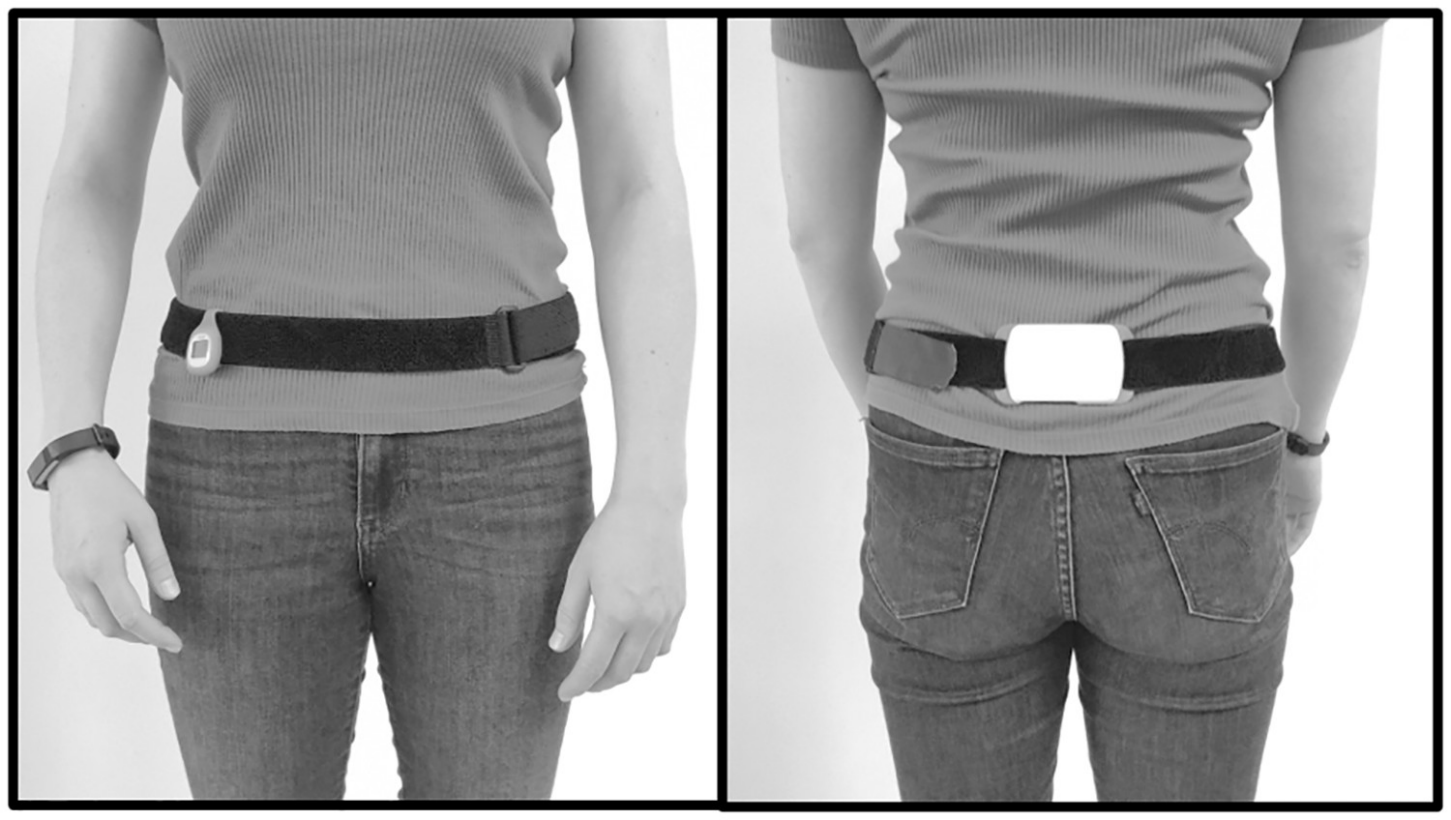
Positioning of the Fitbit Zip (hip), Fitbit Alta (wrist) and Dynaport Movemonitor (lower back).

Patients were provided with an activity monitor (i.e. Dynaport Movemonitor) and two consumer-based activity trackers; i.e. Fitbit Alta, worn at the wrist (wrist-AT) and Fitbit Zip, worn at the hip (hip-AT) (Fig 1). Subjects were instructed to simultaneously wear these three devices for 14 consecutive days during waking hours, except for bathing or showering.

The Dynaport Movemonitor (DAM, McRoberts BV, The Hague, the Netherlands) is a tri-axial accelerometer, which is validated to objectively measure PA in patients with COPD [6, 7]. This monitor is worn at the lower back and does not provide direct feedback. Battery autonomy of this device is 14 days. The DAM captures amount (e.g. expressed as step count) and intensity as well as time in different postures (e.g. standing, lying, walking) and wearing time.

### Physical activity trackers

The Fitbit Zip (hip-AT) (Fitbit, Inc., San Francisco, USA) is a consumer-based activity tracker with clip system, worn at the hip, which we attached on the same strap as the DAM, at the position of the right hip. This device measures daily step count, walking distance and estimates energy expenditure, based on an in-build tri-axial accelerometer and use motion pattern algorithms. Direct feedback on these parameters and the watch function were displayed on the LCD screen of the device. The 3V coin battery was used, with an autonomy of 4 to 6 months.

The Fitbit Alta (wrist-AT) (Fitbit, Inc., San Francisco, USA) is a tri-axial consumer-based activity tracker, which is worn on the wrist. The device was worn at the side preferred by the subject. The device registers daily step count, walking distance, energy expenditure and estimates time spent in moderate to intense activities, based on information from the tri-axial accelerometer and use of motion pattern algorithms. For this observational study, only feedback on daily step count, the watch function and battery level were activated on the 1’4” inch OLED display. Battery charging was necessary every 5 to 7 days and was instructed to all subjects.

Daily step count retrieved from the DAM, hip-AT and wrist-AT and movement intensity during walking captured by DAM were used for the present analyses. The wearing time was only registered by the DAM. Days with a wearing time lower than 8 hours based on DAM were excluded and only days with matching data from all three devices were used in the analyses [22]. Daily step counts of the activity trackers was extracted from the online Fitbit platform at the second clinical visit.

### Participant experience

User friendliness of the hip-AT and wrist-AT was evaluated in a project-tailored questionnaire. Questions were 1) “How pleasant was it to wear this device?”; 2) “How often did you look at the steps displayed on the activity tracker?” and 3) “How long would you like to use this device in the future?”. All questions came with 5 answering possibilities representing a 5 point Likert scale. As final question, subjects were asked to choose which device they would prefer to use in the future (hip worn activity tracker (e.g. Fitbit Zip), wrist worn activity tracker (e.g. Fitbit Alta), both or none of both).

### Statistical analysis

The present study included a convenience sample of 30 patients with COPD, based on similar studies in the field [23] and large enough to cover the whole spectrum of the disease. All statistical analyses were performed using the SAS statistical package (V9.4, SAS Institute, Cary, North Carolina, USA). All data are presented as mean ± SD, unless specified otherwise. Statistical significance was set at p < 0.05 for all analyses. Patients were included in the analyses if they had at least 3 valid days (e.g. > 8 hours of wearing time) with corresponding data with the 3 devices.

First, to investigate the accuracy of the consumer-based activity monitors in capturing PA as a clinical trial endpoint (activity trackers as measuring tool), we 1) compared mean daily step count per patient measured by hip-AT and wrist-AT with DAM (= reference), by use of a paired t-test, in COPD and healthy controls separately. To test the hypothesis that the accuracy of the trackers relates to walking speed, we pooled the groups and tested the association between movement intensity during walking (in quartiles) and the difference between the trackers and the DAM using univariate linear analyses; 2) we compared mean daily step count between COPD and healthy control using an unpaired t-test for every device separately; and 3) we investigated the agreement of mean step count measured by the activity trackers as compared to the DAM by Bland-Altman plots. These analyses were performed for patients with COPD and healthy controls separately.

Second, to evaluate the accuracy of these activity trackers as coaching incentive for an individual subject (activity trackers as coaching tool), we 1) calculated day-by-day differences to the individual mean PA (for example see Fig 3 panel A). This was done for each patient for each of the devices (DAM, hip-AT and wrist-AT). If more than 10 valid days were available, this analysis was based on 10 randomly selected days. Else, all available valid days were used. The day-by-day data were individually sorted based on the DAM measurement, from most active day to least active day and corresponding days with hip-AT and wrist-AT were added to the sorted database. Mean day-by-day differences for each day for each device were calculated and graphically presented; and 2) the step count data retrieved from the three devices were used to evaluate consistency of ranking between DAM and respectively hip-AT and wrist-AT. Daily step count of the selected 10 random days were ranked from most active day to least active day for each device separately. The ranking scores of each day for each device was compared. Consistency of these rankings was evaluated by a Kendall correlation. Correlation coefficient was interpreted using the following cutoffs: weak correlation r = 0.3–0.5; moderate correlation r0.5–0.7; strong correlation r = 0.7–0.9; very strong correlation r>0.9 [24].

Finally, answers on the different questions measuring user preference were compared between the activity trackers using chi-square analyses. Because we hypothesized that the choice of the tracker would depend on the age of the subject [25], we calculated the proportion of older (>65y) and younger (<65y) subjects.

## Results

30 patients with COPD and 15 age-matched healthy controls wore the Dynaport Movemonitor (DAM), Fitbit Zip (hip-AT) and Fitbit Alta (wrist-AT) for 14 consecutive days. Valid data of the three devices could be retrieved in 28 patients with COPD (mean (SD) 11 (3) days of wearing, total of 306 patient-days) and 14 healthy controls (mean (SD) 13.4 (2) days of wearing, total of 188 patient-days), and therefore included in the present analyses. Missing data were due to technical problems with the wrist-AT in two patients with COPD, 1 control subject had insufficient wearing time on all days of testing. Baseline characteristics of the subjects included in the analyses are displayed in Table 1.

**Table 1:** Characteristics of all subjects in the analyses, expressed as mean ± standard deviation, except ◆ median [Q1–3].

	COPD patients (N = 28)	Healthy controls (N = 14)	p-value
**Age (years)**	66 ± 8	69 ± 7	0.15
**Gender (% male)**	61	64	0.72
**BMI (kg/m^2^)**	25 ± 4	27 ± 3	0.05
**FEV_1_ (% pred)**	47 ± 18	122 ± 14	<0.0001
**6MWD (m)**	454 ± 109	660 ± 83	<0.0001
**6MWD (% pred)**	72 ± 15	109 ± 10	<0.0001
CAT (score)*	19 ± 6	*(6* ± *5)*	<0.0001
**mMRC (score)◆**	2 [1–3]	0 [0–0]	<0.0001
C-PPAC (score)*	61 ± 11	*(88 ± 6)*	<0.0001

*CAT and PROactive score only indicative in healthy controls because questionnaire is only validated for use in patients with COPD. P-value based on unpaired t-test or Chi Square. BMI = body mass index; 6MWD = 6-minute walking distance [26]; CAT score = COPD Assessment Test; mMRC = modified Medical Research Council Dysnea Scale (0–4); C-PPAC = clinical visit version PROactive questionnaire—total score (0–100), with higher score indicating better physical activity experience.

### Activity trackers as a measurement tool

In the COPD group, mean step count measured by hip-AT was significantly lower (mean±SD Δ -1112 ± 872 steps per day or -23%; p<0.0001) as compared to DAM, see Table 2. The measurement by wrist-AT was not significantly different (mean±SD Δ +374 ± 1257 steps per day or +8%; p = 0.13). In the healthy controls, the assessment by wrist-AT was significantly higher as compared to DAM (mean±SD Δ +1907 ± 2147 steps per day or +24%; p = 0.006) with no difference between hip-AT and DAM (mean±SD Δ -392 ± 1116 steps per day or +5%; p = 0.21).

**Table 2:** Average step count in patients with COPD and healthy controls. P-value compares average steps measured by Dynaport Movemonitor (DAM) to respectively Fitbit Zip (hip-AT) and Fitbit Alta (wrist-AT), using paired t-test.

	DAM	hip-AT	wrist-AT
**Average daily step count COPD (mean ± SD)**	4785 ± 2560	3673 ± 2332	5158 ± 3020
**p-value compared to DAM (COPD)**		p < 0.0001	p = 0.13
**Average daily step count healthy controls (mean ± SD)**	7811 ± 3215	7419 ± 3406	9718 ± 4324
**p-value compared to DAM (healthy)**		p = 0.21	p = 0.006

The underestimation of the hip-AT to DAM was related to movement intensity during walking, with a smaller difference with higher movement intensity (p-for-trend = 0.016). The accuracy of wrist-AT to DAM was lower with increasing movement intensity (p-for-trend < 0.0001).

Patients with COPD were significantly less active as compared to healthy controls as measured by the three devices (mean±SD Δ 3027 ± 2790, 3746 ± 2728 and 4559 ± 3497 respectively based on DAM, hip-AT and wrist-AT; p<0.05 for all).

**Fig 2:**
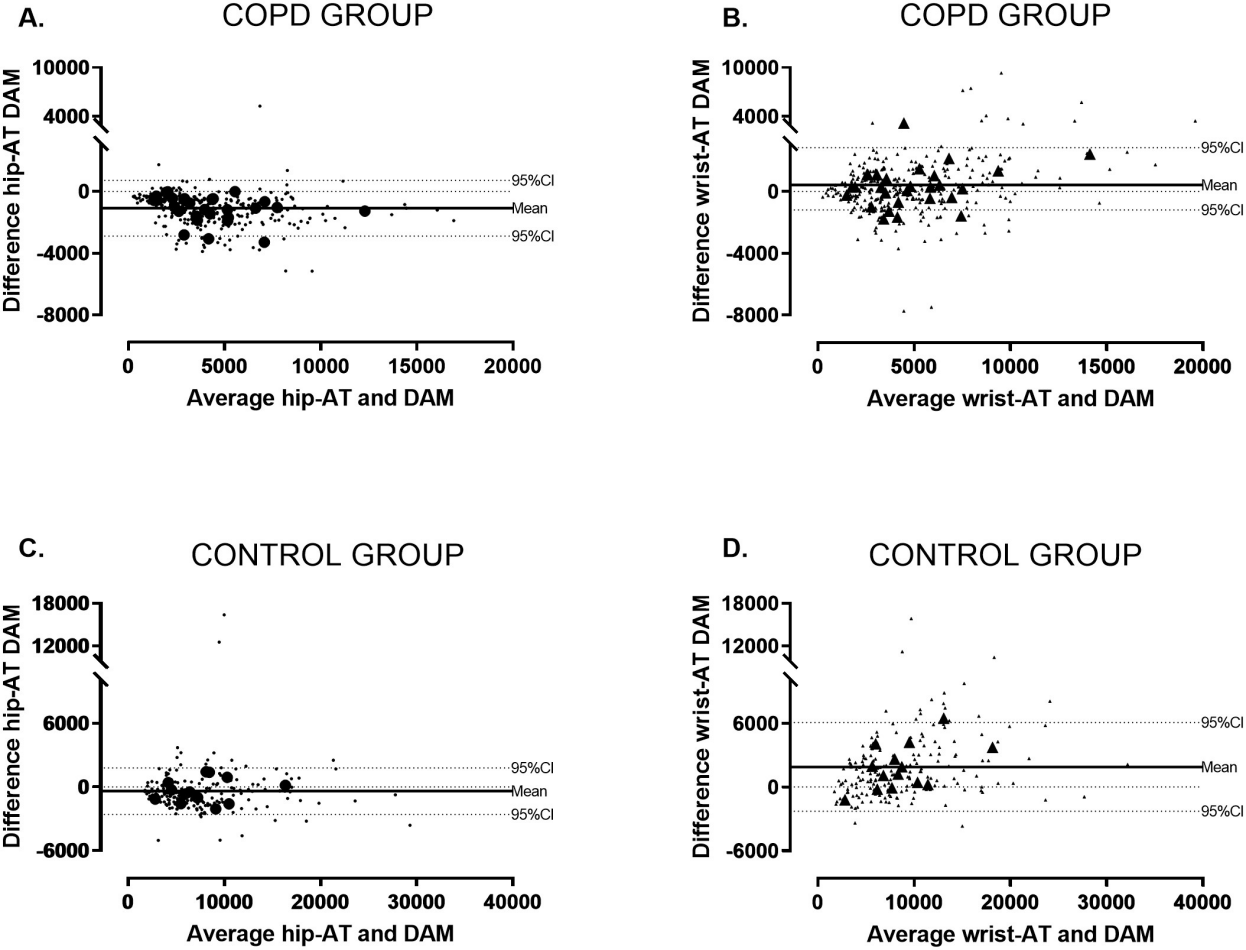
Bland-Altman plots with mean and 95%CI for hip-AT and wrist-AT compared to DAM. (A, B) patients with COPD; (C,D) healthy controls; Large symbols represent the average individual step count per subject in the study (N = 28 for COPD and N = 14 for healthy controls). Small dots represent the daily physical activity expressed as steps per day. Mean and 95%CI are calculated based on average step count data. Hip-AT = Fitbit Zip; wrist-AT = Fitbit Alta; DAM = Dynaport Movemonitor.

The Bland-Altman analysis presented in Fig 2 (mean step count, large dots) shows a lower mean bias for PA measured by hip-AT in healthy subjects as compared to patients with COPD (respectively bias (95%CI) for healthy controls and COPD: -391 (-2579; 1797) steps and -1055 (-2820; 589) steps). The bias measured by wrist-AT was larger in healthy controls as compared to patients with COPD (respectively bias (95%CI) for healthy and COPD: 1891 (-2286; 6068) steps and 306 (-2068; 2680) steps).

### Activity trackers as a coaching tool

**Fig 3:**
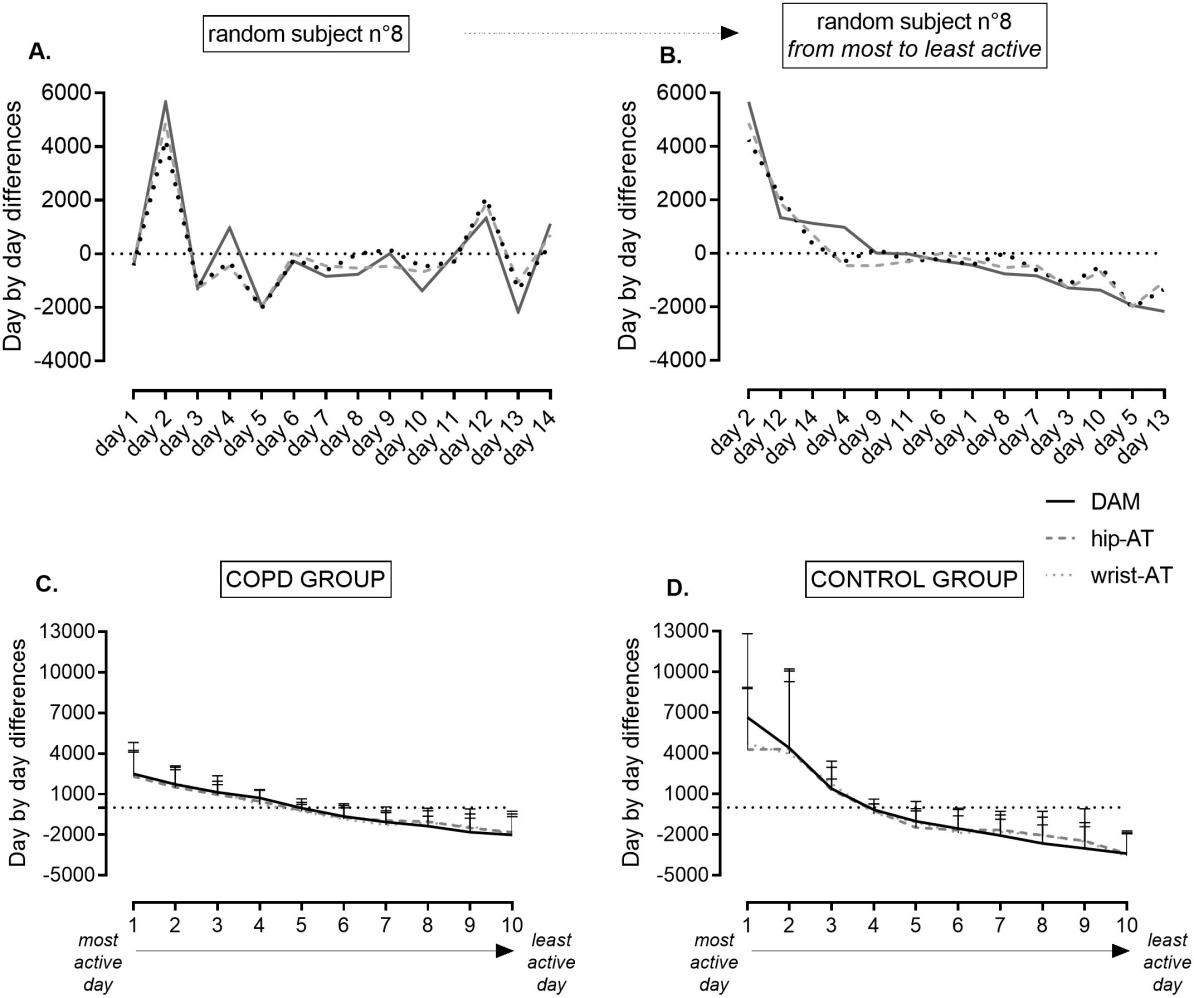
Graphical representation of day-by-day variability in PA, expressed as steps per day around the individual mean PA. Panel A: day-by-day differences around the mean step count of one random patient, recorded by hip-AT, wrist-AT and DAM. Horizontal dotted line represents the mean step count, positive numbers representing more active days as compared to the mean PA measured, negative numbers presenting less active days as compared to the mean PA. Panel B: Individual data (1 random patient) of panel A were ranked from the most active day to the least active day according to DAM. Corresponding data of hip-AT and wrist-AT were added. Finally, after ranking the data for every individual subject, mean (±SD) were calculated for the COPD group and healthy control group. Pooled data are represented in panel C (COPD group) and panel D (control group). Hip-AT = Fitbit Zip; wrist-AT = Fitbit Alta; DAM = Dynaport Movemonitor.

Fig 3 shows the day-by-day variability expressed as mean day-by-day differences measured by the three devices. Difference to the individual mean PA followed the same pattern measured by DAM, wrist-AT and hip-AT. Both activity trackers have the ability to detect patterns of more and less active days similarly to the DAM, both in COPD (panel C) and healthy controls (panel D).

**Fig 4:**
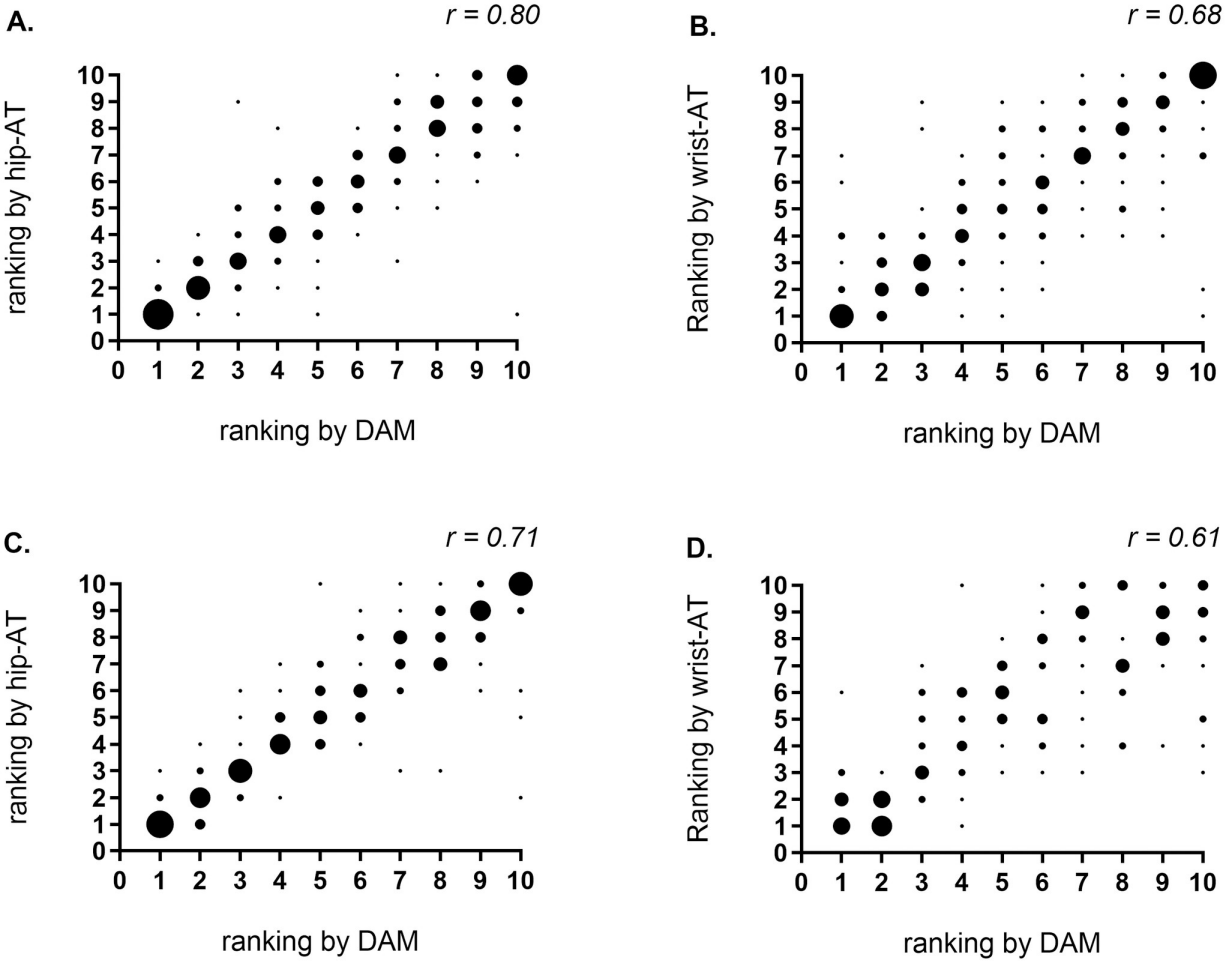
Ranking of daily step count by DAM compared to ranking by activity trackers. DAM compared to hip-AT in COPD (panel A) and healthy controls (panel C) and compared to ranking by wrist-AT, in COPD (panel B) and healthy controls (panel D). The larger the dot, the larger number of subjects for the given combination of ranks, r = Kendall correlation for consistency of ranking. Hip-AT = Fitbit Zip; wrist-AT = Fitbit Alta; DAM = Dynaport Movemonitor.

Finally, consistency of ranking from most active to less active days showed a moderate to strong correlation in the COPD group for both hip-AT and wrist-AT (Kendall correlation respectively 0.80 and 0.68). In the healthy controls, correlation for hip-AT and wrist-AT were respectively 0.71 and 0.61, see Fig 4.

### User preferences

54% of the COPD patients preferred the wrist worn device; a majority (69%) of these subjects preferring wrist-AT were younger than 65 years old. 42% of the COPD group preferred the hip worn device, which were mostly older patients (70% of the subjects preferring the hip worn device were older than 65 years). One patient preferred none of both devices. In healthy subjects, 50% of the healthy subjects preferred wrist-AT, whereas 21% preferred hip-AT. Two subjects chose both devices. Preferences in healthy controls were not related to age.

Detailed information on the user experience is displayed in Table 3. The majority of subjects in both the COPD group and control group scored the activity trackers as pleasant or very pleasant. Only a minority of subjects in the COPD group scored the hip-AT and wrist-AT as unpleasant.

**Table 3:** User experience, expressed as percentage, based on project-tailored questionnaire for the hip worn activity tracker (hip-AT) and wrist worn activity trackers (wrist-AT) in COPD and control group.

		COPD		Control	
		Hip-AT	Wrist-AT	Hip-AT	Wrist-AT
***How pleasant was it to wear the tracker***?	Very pleasant / pleasant (%)	40	54	57	57
	Neutral (%)	46	25	43	43
	Not pleasant (%)	14	21	0	0
***How often did you look at the steps on the tracker***?	Few times per day / once a day (%)	61	82	58	79
	Sometimes, not daily (%)	14	7	14	14
	Once / twice a week (%)	4	0	7	7
	Never (%)	21	11	21	0
***How long would you like to wear the tracker in the future***?	Few months / more than 1 year (%)	21	40	32	58
	Few days / few weeks (%)	58	42	53	21
	Never again (%)	21	18	15	21

Subjects in the COPD group reported that they looked more often to the wrist-AT as compared to the hip-AT (hip-AT vs wrist-AT: p = 0.02; p = 0.65 respectively in COPD and controls). Small differences in favor of wrist-AT could be observed for future use, however no significant differences between hip-AT and wrist-AT could be found for future use and pleasantness in the COPD and control group.

## Discussion

The present paper shows that 1) to capture physical activity as clinical trial endpoint, the hip worn activity tracker (hip-AT, Fitbit Zip) significantly underestimates daily step count in patients with COPD. This underestimation is less prominent in healthy subjects. The wrist worn activity tracker (wrist-AT, Fitbit Alta) showed a non-significant overestimation of step count in patients with COPD, and a significant overestimation of daily steps in healthy age-matched controls; 2) as a coaching tool, both the hip and wrist worn activity tracker were able to pick up the day-by-day variability as measured by DAM. Additionally, a good consistency of ranking of days was found for both the hip and wrist worn activity monitor compared to DAM in patients with COPD; 3) finally, overall preference was fairly similar for both devices, although an age difference could be observed in patients with COPD (e.g. higher proportion of younger subjects preferred the wrist worn device, higher proportion of older subjects preferred the hip worn device).

### Comparison with other studies

According to Tudor-Locke et al., less than 10% error in the physical activity measure is acceptable for activity trackers in free-living conditions [27]. Based on these criteria, step count recorded by hip worn devices in healthy subjects and wrist worn devices in COPD subjects would be acceptable to measure PA. However, we believe that caution is warranted when using these activity trackers as measurement tool or physical activity endpoint for trials. The significant underestimation of step counts in patients with COPD could be explained by the decreased walking speed of this group, based on the lower 6MWD in patients as compared to the healthy controls. Previous studies investigated the influence of walking speed on accuracy of hip-worn activity trackers in laboratory settings. They showed a significant underestimation ranging from 23% to 67% error in waist-worn devices at slower walking speeds in controlled settings [9, 10, 28]. Indeed, our results showed a significant association between movement intensity during walking and the accuracy of the hip trackers using free-living data (i.e. higher underestimation with lower movement intensity).

A recent systematic review on the accuracy of Fitbit devices endorses our findings that activity trackers tend to overestimate daily step count during free-living circumstances in healthy subjects [23]. In our group, this overestimation of wrist-worn devices was significantly larger in healthy subjects as compared to patients with COPD. This could possibly be explained by the smaller spectrum of daily life activities in patients with COPD. This pattern can also be seen in the Bland Altman plot, showing a larger difference in patients doing more activity, and the significant negative association between movement intensity during walking and accuracy. We hypothesize that wrist-worn activity trackers incorrectly classify non-walking activities more easily as step-like movements. As patients with COPD perform less daily life activities (average step count for respectively healthy and COPD: 7811 ± 3215 steps/day vs 4785 ± 2560 steps/day), the error percentage is smaller as compared to healthy individuals. However, with the current data we can only speculate on the mechanism explaining the differences in step count between both groups. Real-time minute-by-minute step count data is necessary to confirm this suggestion. Floegel et al. showed an underestimation of step count by wrist worn devices in older subjects using a cane or walker [28]. In the current study, we excluded subjects with walking aids or abnormal gait pattern but it can be advised to use hip worn devices in patients who use walking aids.

Lower cost and widespread availability of consumer-based activity trackers can make these devices attractive options to use in different settings. However, other points of attention when considering the use of step counters as an outcome measure, that are outside our current research question, are first that the outcome is limited to the number of steps per day. This omits movement intensity as an important feature of physical activity. Second, wearing time is generally not provided with activity trackers. In our study, we only used days with sufficient wearing time with the gold standard, but most step counters (including Fitbit products) and smartphone applications do not report wearing time. Nevertheless, this is important to consider in studies of effectiveness [22].

To the best of our knowledge, the accuracy of these activity trackers as coaching tool in patients with COPD has not been investigated before. We believe that with the present statistical approach, we prove that activity trackers provide the information required in a coaching intervention (e.g. distinguishing more from less active days in an individual subject) in a practical way and that they can be used in physical activity coaching interventions. The use of hip worn physical activity trackers has shown to be effective to improve physical activity in previous coaching trials [16, 29]. Subjects in coaching interventions should be instructed to wear the device during waking hours. As indicated before, unfortunately quality of wearing time is not possible with most of the devices.

User preferences in our cohort concur with previous research in an Australian healthy population, which revealed slightly higher proportion of preference towards wrist worn devices [25]. We found that older patients with COPD (> 65 years) had a higher preference for hip worn device, whereas younger patients with COPD (< 65 years) preferred a wrist worn device more. When aiming for long-term behavior change and using these activity trackers as coaching tool, preference of the user should be taken into account to optimize long-term compliance. Such interventions should allow user preference in the coaching device of choice. We found that a small proportion of the subjects (1 patient with COPD and 2 healthy subjects) preferred none of both devices. We need to keep in mind that these activity trackers are not ‘one fits all solutions’ and that other interventions independent of such activity tracker are necessary.

### Strengths & limitations

By using these specific statistical approaches, we were able to identify day-by-day variability of the different devices and compare the consistency of ranking more to less active days by the different devices. With this approach, we showed that activity trackers can be used as a coaching tool. By comparing activity monitoring in both a chronic diseased population and healthy age-matched subjects and capturing data in free-living conditions, we attempted to provide evidence across a wide range of daily life physical activity behavior.

Although our research provided unique insights in the accuracy of activity trackers as measuring and coaching tool, the following limitations should be taking into account. In this study, Dynaport MoveMonitor (DAM) served as a criterion standard, allowing the investigation of the activity trackers in free-living conditions. Although the activity monitor (DAM) is valid to measure PA in patients with COPD, there might still be a slight deviation from video recording or manual step counting by a researcher. Due to the free-living setting, the use of these gold standard methods were not possible. Finally, one should be cautious with extrapolating the present findings to all consumer-based activity trackers. However, as our results are in line with previous studies using different activity trackers [9, 23, 28], it is likely to find similar results with other consumer-based activity trackers.

## Conclusion

Our research confirms that popular activity trackers (Fitbit) lack the accuracy to capture physical activity endpoints in clinical trials, especially hip worn devices in a slow walking population and wrist worn devices in a more active population. To measure physical activity, using more sophisticated and properly validated monitors are required.

Nevertheless, the present study provides new evidence on the validity of consumer-based activity trackers as coaching tool. Our results confirm that these activity trackers are valid to use as a coaching tool in both healthy and chronic diseased subjects.

## Abbreviations

6MWD: Six-Minute Walk Distance
BMI: Body Mass Index
CAT: COPD Assessment Test
COPD: Chronic Obstructive Pulmonary Disease
C-PPAC: PROactive Questionnaire–clinical visit version
DAM: Dynaport MoveMonitor
FEV_1_: Forced Expiratory Volume in the first second
FVC: Forced Vital Capacity
Hip-AT: hip worn activity tracker
mMRC: modified Medical Research Council
PA: Physical Activity
Wrist-AT: wrist worn activity tracker
